# Repurposing Approved Drugs for Sarcopenia Based on Transcriptomics Data in Humans

**DOI:** 10.3390/ph16040607

**Published:** 2023-04-18

**Authors:** Shuang Liang, Danyang Liu, Zhengwu Xiao, Jonathan Greenbaum, Hui Shen, Hongmei Xiao, Hongwen Deng

**Affiliations:** 1 Center for System Biology, Data Sciences, and Reproductive Health, School of Basic Medical Science, Central South University, Changsha 410013, China; 2 Laboratory of Molecular and Statistical Genetics, College of Life Sciences, Hunan Normal University, Changsha 410013, China; 3 Tulane Center of Biomedical Informatics and Genomics, Deming Department of Medicine, Tulane University School of Medicine, New Orleans, LA 999039, USA

**Keywords:** sarcopenia, drug repositioning, weighted correlation network analysis (WGCNA), gene set enrichment analysis (GSEA), differential analysis

## Abstract

Sarcopenia, characterized by age-related loss of muscle mass, strength, and decreased physical performance, is a growing public health challenge amid the rapidly ageing population. As there are no approved drugs that target sarcopenia, it has become increasingly urgent to identify promising pharmacological interventions. In this study, we conducted an integrative drug repurposing analysis utilizing three distinct approaches. Firstly, we analyzed skeletal muscle transcriptomic sequencing data in humans and mice using gene differential expression analysis, weighted gene co-expression analysis, and gene set enrichment analysis. Subsequently, we employed gene expression profile similarity assessment, hub gene expression reversal, and disease-related pathway enrichment to identify and repurpose candidate drugs, followed by the integration of findings with rank aggregation algorithms. Vorinostat, the top-ranking drug, was also validated in an in vitro study, which demonstrated its efficacy in promoting muscle fiber formation. Although still requiring further validation in animal models and human clinical trials, these results suggest a promising drug repurposing prospect in the treatment and prevention of sarcopenia.

## 1. Introduction

The global population is ageing rapidly. Following data reported in the United Nations *World Population Ageing 2015*, from year 2015 to 2050, the global population aged 60 years and above will increase by nearly twofold, reaching 2.1 billion people [1]. Ageing brings a range of profound changes in body composition, especially in skeletal muscle function and mass, which is a major risk factor for sarcopenia. Sarcopenia, an age-associated disease characterized by diminished skeletal muscle strength and mass, and low physical performance [2], is significantly related to an augmented probability of falls, hospitalization, frailty, morbidity, and mortality in the elderly. Recent studies conducted in the United States and the United Kingdom anticipated that individuals who develop sarcopenia might experience higher healthcare costs than those who are unaffected. In the UK, the mean annual cost per person was estimated to be £1885 for individuals without muscle weakness, and £4592 for individuals with muscle weakness [3]. Similarly, sarcopenia-caused hospitalizations and other healthcare costs amounted to an estimated $2315.7 more per person, per year in the United States than for individuals without sarcopenia [4]. This poses a substantial burden on healthcare resources and carries a significant economic impact in society. Therefore, in recent years, greater attention has been dedicated to sarcopenia.

At present, there are no approved medications to treat sarcopenia. Based on the existing clinical studies, exercise was proven to be the most effective intervention for alleviating sarcopenia [5]. Other interventions also exhibit some therapeutic benefits, including nutritional supplementation, medications, and physiological intervention [6,7]. However, all these treatments suffer from their respective limitations [5,6,8]. Adherence to regular exercise regimens can be challenging for the elderly population. Individuals with physical frailties cannot perform effective exercise routines and nutritional supplementation does not exhibit significant improvement towards physical performance and muscle strength [6,8]. Although a few promising drugs have been developed, they oftentimes exhibit several adverse side effects, obstructing their widespread use. For example, growth hormone (GH) may induce fluid retention, orthostatic hypotension, and cancer induction; while testosterone may induce fluid retention, gynaecomastia, worsening of sleep apnoea, polycythaemia, and acceleration of benign or malignant prostatic disease [5]. Therefore, the search for reliable therapeutic options for sarcopenia is imperative and becoming increasingly important.

However, the drug discovery and development procedure is not only expensive but also time-consuming, with low success probability in drug approval. Consequently, through drug repurposing identifying new therapeutic approaches has garnered increasing attention in recent years. For instance, during the early period of the COVID-19 pandemic, researchers found that dexamethasone could improve outcomes for patients with mild or severe COVID-19 [9]. Compared to traditional drug development, drug repurposing is a promising method to find effective drugs in less time and at a lower cost. Accordingly, a number of computational methods have been developed for drug repurposing [10]. However, due to the complexity of the disease and the human drug response, correlating effective medications to a disease with the available information of drugs (3D chemical structure, target, side effects, transcriptome profiles of drug, etc.) and disease (electronic health record, multi-omics data, etc.) can be challenging. Researchers commonly use a combination of multiple computational methods to perform drug repurposing to increase the reliability of the results. For instance, Misselbeck et al. used network proximity and semantic similarity to identify drug effects in metabolic syndrome [11], while Cheng et al. integrated network proximity and gene set enrichment analysis (GSEA) to identify more efficacious therapeutic selections for multiple cancer types [12]. Morselli Gysi et al. used artificial intelligence, network diffusion, and network proximity to identify promising drug candidates for COVID-19 [13]. The results of Morselli Gysi et al. study indicated that no single method was superior to others, highlighting the necessity for an ensemble approach that integrates various data sources. In the future, with the accumulation of various study data, greater importance will be placed on the effective integration of various data sources.

In our study, we performed an integrative drug repurposing analysis to repurpose previously approved drugs for sarcopenia. The results of our study indicated that the first-ranked drug vorinostat is a promising candidate to treat sarcopenia based on in vitro validation experiments.

## 2. Results

### 2.1. Overview of the Analysis Process

**Figure 1:**
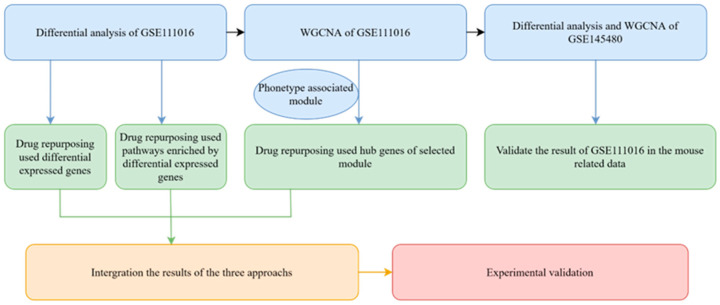
Flow diagram of the drug repurposing analysis. Step 1: Differential analysis, ranked gene set enrichment analysis, and WGCNA of GSE111016 and GSE145480. Step 2: Three different algorithms were used to perform drug repurposing, respectively, based on the differentially expressed genes, significant enrichment pathways, and hub genes of the co-expression module. Step 3: CRank algorithm was used to integrate the results of the three different drug repurposing algorithms. Step 4: Top-ranked vorinostat was selected for experimental validation in vitro.

With the integrated drug repurposing approach, our article seeks to repurpose approved drugs for sarcopenia based on transcriptome data. As shown in Figure 1, the analysis pipeline can be divided into four sections. The first step focuses on the differential analysis, ranked gene set enrichment analysis, and weighted gene co-expression network analysis (WGCNA) of the GSE111016 dataset which includes 20 sarcopenia patients and 20 controls. To verify the results derived in GSE111016, differential analysis and WGCNA were also conducted using transcriptome data from mouse gastrocnemius of different ages (GSE145480). Second, drug repurposing was performed using three different algorithms based on the differentially expressed genes, significant enrichment pathways, and hub genes of the co-expression module. The CRank algorithm was used to integrate the three results [14]. This approach was chosen based on its ability to extract the cumulative predictive power of all methods, matching or exceeding the predictive power of each pipeline individually [13]. Finally, the top-ranked drug vorinostat was selected for experimental validation in vitro.

### 2.2. Gene Signature of Sarcopenia Based on the Transcriptomic Differential Analysis in Humans

**Figure 2:**
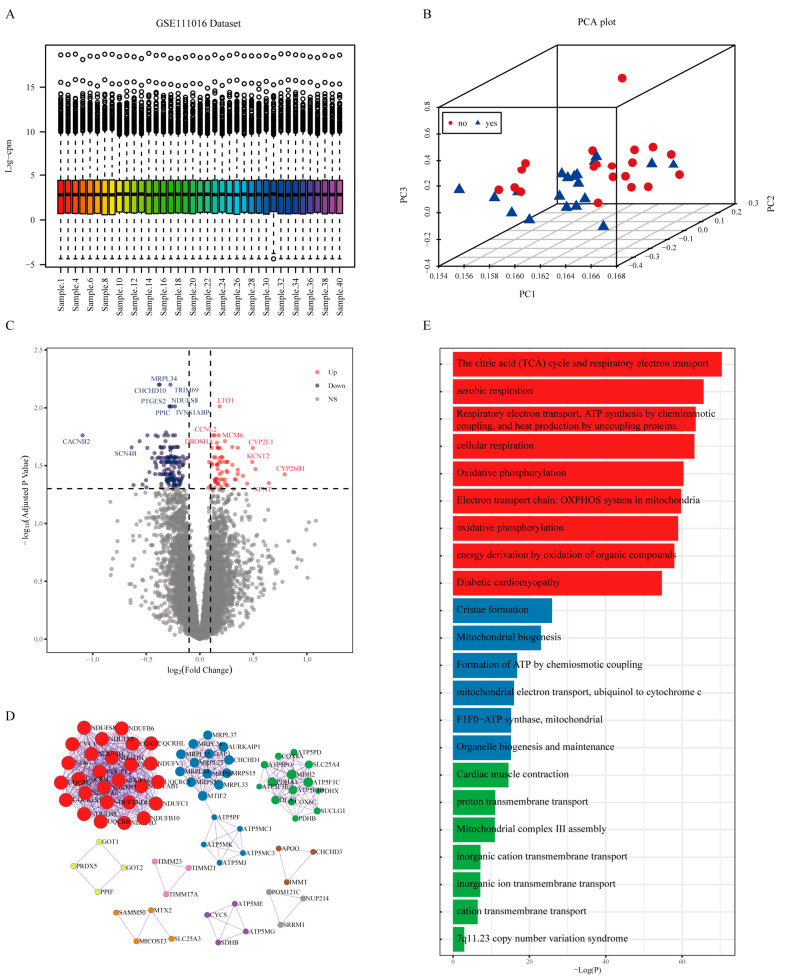
(**A**) Raw count quality control assessment of GSE111016 Dataset. (**B**) Principal component analysis (PCA) of GSE111016. (**C**) A volcano plot of the differential expression analysis of GSE111016. (**D**) The enrichment analysis of differentially expressed genes led to the extraction of 9 sub-networks by Metascape [16]. (**E**) Pathway enrichment analysis of the top three sub-networks.

**Figure 3:**
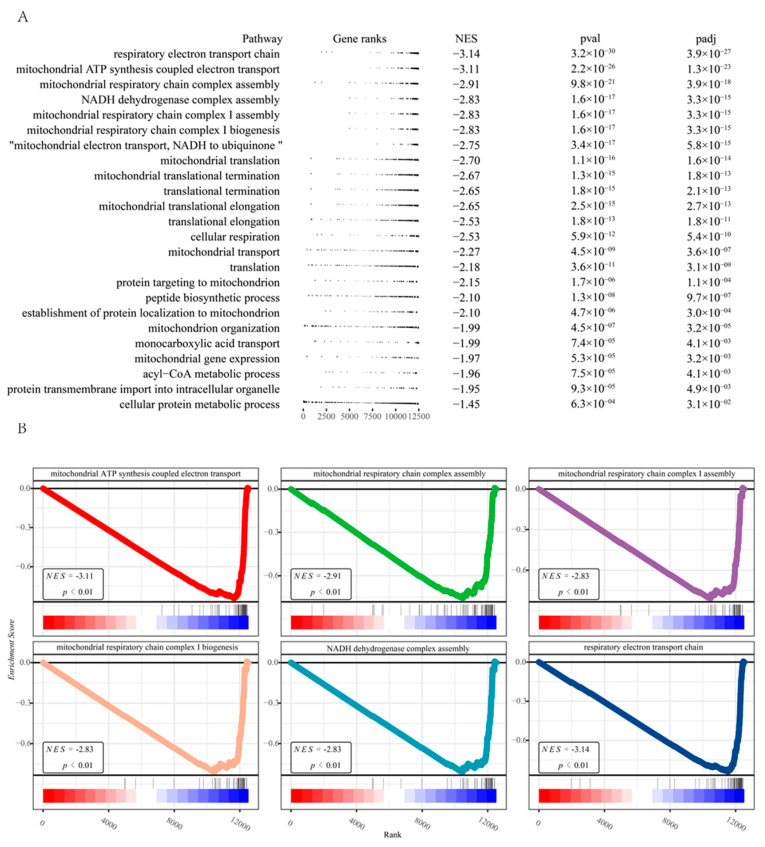
(**A**) A table plot for the ranked GSEA of the differential expression analysis results. (**B**) Enrichment plots of the six top pathways derived from the ranked GSEA.

To obtain the gene signature of sarcopenia, we first conducted a differential analysis of the RNA-seq transcriptome from the human dataset (GSE111016) [15]. Quality control on the raw counts was conducted to ensure the comparability between sarcopenic and healthy subjects (Figure 2A). Principal component analysis (PCA) plots revealed relatively visible differences between the control and sarcopenia groups (Figure 2B). A total of 222 genes (Table S1) were identified with an adjusted *p*-value < 0.05, including 66 up-regulated genes and 156 down-regulated genes (Figure 2C). A protein-protein interaction enrichment analysis was performed using Metascape [16] (http://metascape.org (accessed on 2 July 2021)) on the list of differentially expressed genes. Nine sub-networks were extracted with the MCODE [17] algorithm as shown in (Figure 2D). Pathway enrichment analysis of the top three sub-networks showed that the citric acid (TCA) cycle and respiratory electron transport (*p*-value = 3.88 × 10^−71^), aerobic respiration (*p*-value = 2.24 × 10^−66^), and oxidative phosphorylation (*p*-value = 1.06 × 10^−59^) were significantly enriched (Figure 2E). We also found that the enrichment results of the three sub-networks are closely related to mitochondrial function, aligning with previous research [18,19]. Following the differential expression analysis of the GSE111016, we ordered the gene list based on their log fold change and performed a ranked GSEA [20]. Results from the ranked GSEA (Figure 3) indicated that mitochondria-associated pathways were significantly reduced in sarcopenia patients. According to the results of the ranked GSEA, mitochondrial dysfunction plays a significant role in sarcopenia.

### 2.3. Gene Signature of Sarcopenia Based on the WGCNA

**Figure 4:**
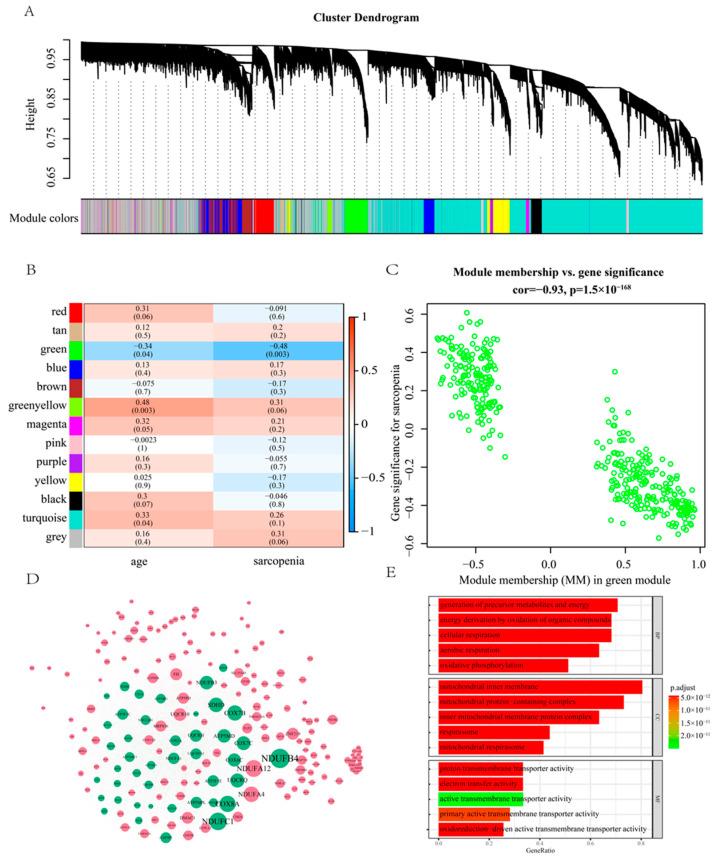
(**A**) Cluster dendrogram of the 39 samples in GSE111016. (**B**) Heatmap of the correlation between the module eigengenes and phenotype. (**C**) Visualization of gene significance (GS) vs. module membership (MM) and gene expression levels of the green module. (**D**) Co-expression network of the green module. The green dots represent the significantly differentially expressed genes. The size of the dots stands for the absolute value of the log fold change. (**E**) Gene Ontology enrichment analysis of the 43 significant genes (Section 2.4).

WGCNA is an effective tool for understanding how genes jointly impact complex human diseases. The hub genes in co-expressed gene modules could be potential as disease markers or therapeutic targets. Within our study, we detected 12 gene modules with the dynamic tree-cutting algorithm (Figure 4A). The green module exhibited the most negative correlation with sarcopenia and age as well (Figure 4B,C). The green module comprises 385 genes (Figure 4D, Table S2). Gene set enrichment analysis was performed on genes within the green module that met the following criteria: (1) module membership (MM) more than 0.7; (2) gene significance (GS) less than −0.4 with sarcopenia; (3) adjusted *p*-value for differential expression analysis is less than 0.05 (Section 2.2). Module membership (MM) is a statistical measure of the correlation between gene expression and each module eigengene. The module eigengene was defined as the first principal component of the module. Gene significance (GS) is the correlation between gene expression and each trait. Finally, 43 significant genes were included in a Gene Ontology enrichment analysis (Figure 4E). We found the 43 genes were mainly enriched in cellular respiration (adjust *p* value = 3.13 × 10^−42^), oxidative phosphorylation (adjust *p* value = 9.07 × 10^−33^), mitochondrial protein-containing complex (adjust *p* value = 4.33 × 10^−48^), and electron transfer activity (adjust *p* value = 1.83 × 10^−48^).

### 2.4. Differential Analysis and WGCNA of Mouse Gene Expression Data

**Figure 5:**
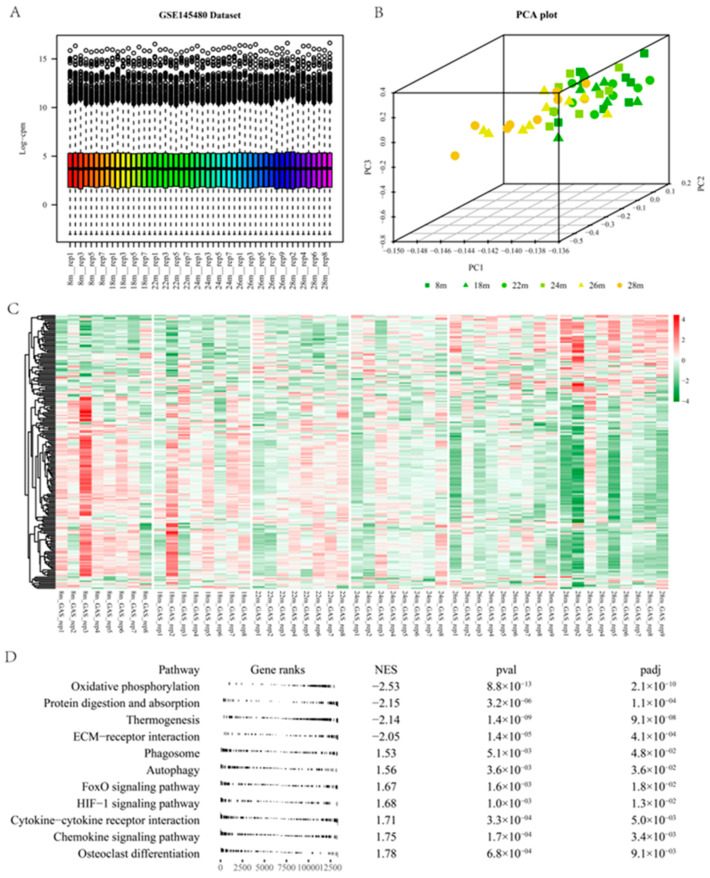
(**A**) Raw count quality control assessment of GSE145480 Dataset. (**B**) Principal component analysis (PCA) of GSE145480. With the progressive ageing of mice, there is a discernible modification to the gene expression profile of the skeletal muscle tissue. (**C**) Heatmap of the 211 mouse genes (orthologous genes of the differentially expressed genes in GSE111016) in the GSE145480. (**D**) A table plot for the Ranked GSEA of GSE145480 based on the mouse KEGG pathways set.

Aged mice are commonly used as models for sarcopenia [21]. Gene expression data from skeletal muscle of mice were used to identify critical genes associated with skeletal muscle function. Phenotypic analysis of the GSE145480 revealed that grip strength and muscle cross-sectional area decreased with advancing age [22]. Quality control was also performed on the GSE145480 (Figure 5A). PCA exhibited the separation of the old group from the young group (Figure 5B). Two hundred twenty-two differentially expressed genes in GSE111016 were converted into 211 mouse orthologous genes in the GSE145480. A heatmap of their relative expression values at different ages was plotted (Figure 5C). The expression levels of up-regulated mouse orthologs in the GSE111016 indicated an increasing trend as the mice aged. Down-regulated mouse orthologs showed an opposite trend as the mice aged. These results further confirmed the association of differentially expressed genes in GSE111016 and sarcopenia. The complete gene list of GSE145480 was ranked according to their log fold changes (28 m vs. 8 m mouse). The ranked GSEA was performed on the pre-ranked gene list of GSE145480 using the *fgsea* package [20] with KEGG pathways sets of the mouse. The most significantly decreased pathway was Oxidative phosphorylation pathways (NES = −2.53, Figure 5D), indicating the critical role of mitochondrial function in the maintenance of skeletal muscle function.

**Figure 6:**
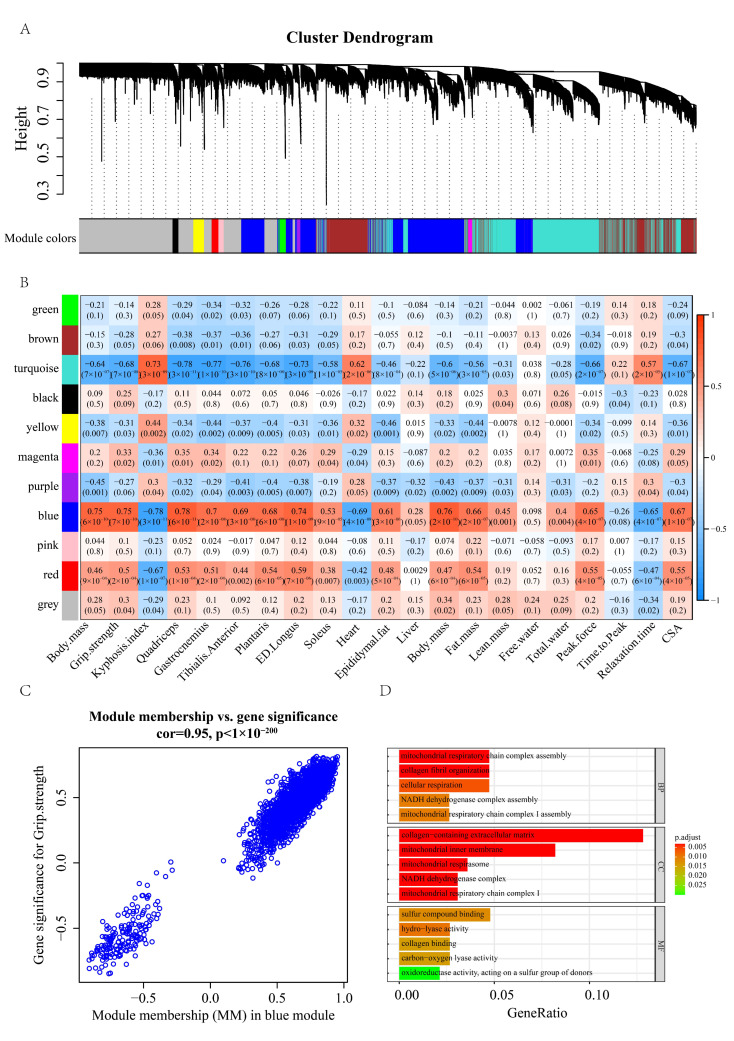
(**A**) Cluster dendrogram of the 50 mouse samples in GSE145480. (**B**) Heatmap of the correlation between the module eigengenes and mouse phenotype. (**C**) Visualization of gene significance (GS) vs. module membership (MM) and gene expression levels of the green module. (**D**) Gene ontology enrichment analysis of the 43 significant genes (Section 2.4).

We proceeded to perform WGCNA on the GSE145480 dataset and identified 10 modules (Figure 6A). The module-trait relationship (Figure 6B,C) showed that blue module has a highly positive relationship with grip strength and CSA (cross-sectional area). We chose the genes with a module membership (MM) greater than 0.7 and gene significance (GS) with grip strength greater than 0.7 for Gene Ontology enrichment analysis (Figure 6D). We observed similar pathway enrichment results to these results in humans (Section 2.4). For example, several mitochondrial functional pathways, such as mitochondrial respiratory chain complex assembly, mitochondrial respiratory chain complex I assembly, and mitochondrial inner membrane, were strongly enriched for the significant genes. The differential analysis and WGCNA results obtained in mouse muscle mRNA profiles further bolstered our findings in the sarcopenic population.

### 2.5. Transcriptome-Based Drug Repurposing

**Figure 7:**
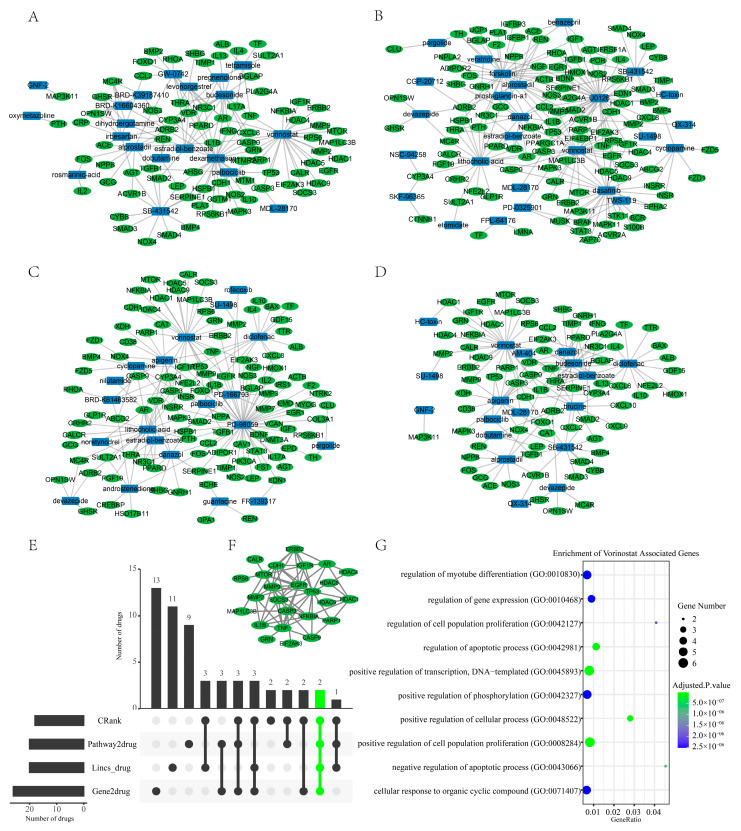
(**A**–**D**): In the top 50 drugs of the four different approaches, we showed the intersection of each with genes associated with sarcopenia ((**A**): Lincs algorithm; (**B**): Gene2drug algorithm; (**C**): Pathway2drug algorithm; (**D**): Crank algorithm). There is no display of drugs that do not interact with sarcopenia-associated genes. (**E**): The UPSet plot shows the intersections of the drugs/small molecules from the four rank lists that have intersected with the sarcopenia-associated genes. (**F**): PPI network of the vorinostat-associated genes. (**G**): Gene Ontology (biological process) enrichment analysis for the 25 genes that are included in the PPI network of vorinostat.

Reversing disease-related expression profiles is a commonly used drug repurposing approach [23,24]. If medications possess therapeutic effects, drug signatures are often presumed to exhibit an inverse correlation with disease signatures. Initially, the *LINCS* algorithm [25] was used to perform drug repurposing with the up- and down-regulated genes. The outcome of the *LINCS* algorithm was presented in Table S3. For a better grasp of the association between sarcopenia and the most highly ranked drugs/small molecules, we presented the intersection of genes that are associated with sarcopenia and drugs/small molecules. The genes related to sarcopenia (Table S4) were obtained from the Open Targets [26] and the DisGeNET database [27]. Open Targets, an open-source tool, utilizes human genetics and genomics data to aid researchers in identifying and giving priority to prospective drug targets for further examination. The DisGeNET platform hosts one of the most extensive publicly available assemblages of genes and variations involved in human diseases. This platform amalgamates information from curated databases, GWAS catalogs, scientific literature, and animal models. The genes associated with drugs/small molecules were downloaded from the STITCH [28] database (combined score > 300). STITCH is a database that encompasses both ascertained and predicted interactions between chemicals-protein or chemicals-chemicals. Literature, databases, and experiments provide evidence linking chemicals to another chemical or protein. Within the top 50 drugs of the LINCS algorithm, we found 20 chemicals have intersections with genes related to sarcopenia (Figure 7A), such as vorinostat, estradiol-benzoate, dexamethasone, SB-431542, and budesonide.

Next, we leveraged the gene2drug [29] computational tool as a second pipeline. Forty-three significant down-regulated genes derived in the intersection of WGCNA and differential analysis (Section 2.4) were used to perform drug repurposing. The result of the second pipeline was presented in Table S5. Among the top 50 drugs identified by the gene2drug algorithm, 26 chemicals have intersections with genes related to sarcopenia (Figure 7B), such as diclofenac, danazol, estradiol-benzoate, dasatinib, and vorinostat.

Furthermore, the pathway-based drug repurposing algorithm offered the third pipeline. We employed the 24 down-regulated pathways (Figure 3A) to perform enrichment analysis for drug repurposing. The results of the pathway-based drug repurposing algorithm were presented in Table S6. Among the top 50 drugs identified by the pathway-based algorithm, 20 drugs have intersections with genes related to sarcopenia (Figure 7C), such as androstenedione, diclofenac, danazol, and vorinostat.

Finally, we used the CRank algorithm [14] to aggregate the three pipelines (Table S7). Among the top 50 drugs identified by the CRank algorithm, 18 drugs have intersections with genes related to sarcopenia (Figure 7D), such as danazol, budesonide, diclofenac, vorinostat, and estradiol-benzoate. The UPSet plot shows the intersections of the four different rank lists (Figure 7E). Noticeably, vorinostat and estradiol-benzoate were identified as top candidates by all four algorithms. The top-ranked vorinostat was selected for further study. We mapped vorinostat-associated genes onto the STRING [30] database to predict the protein-protein interaction network. The vorinostat-related PPI network comprised 25 genes (Figure 7F, interaction score > 0.7). Gene ontology pathway enrichment analysis of the 25 genes indicates that regulation of myotube differentiation (GO:0010830, Adjust *p*-value: 2.53 × 10^−6^) and regulation of cell population proliferation (GO:0042127, Adjust *p*-value: 2.01 × 10^−6^) were significantly enriched (Figure 7G). These results suggested that vorinostat could be a potential candidate for the treatment of sarcopenia, thus we selected vorinostat for further experimental validation.

### 2.6. Literature and Experimental Validation of Drug-Repurposing Results

**Figure 8:**
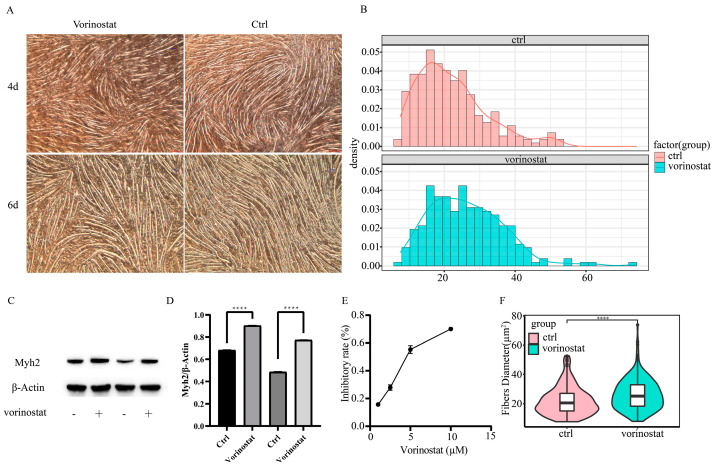
(**A**) 1µM of vorinostat increased the diameter of muscle fibers. (**B**) Density plot of myotube diameter distribution. (**C**,**D**) WB experiments were independently replicated two times. Repeated WB experiments confirmed that the drug intervention group (vorinostat: 1µM) had a higher myotube percentage. (**E**) The cell activity assays (CCK-8) indicated that vorinostat (1 µM, 2.5 µM, 5 µM, 10 µM) inhibits the activity of C2C12 cells. (**F**) Violin plots showing the different diameter distribution in the control vs. vorinostat group (****: *p* < = 0.0001).

To corroborate the results suggested by the integrated algorithm, we selected the top 20 drugs/small molecule compounds for literature review. Five drugs have been suggested as potentially effective treatments for sarcopenia in previous studies (Table 1). These findings lend support to the accuracy of our drug repurposing algorithm to some extent. The top-ranked approved drug vorinostat is an inhibitor of histone deacetylase (HDAC). The prior study has demonstrated that the suppression of HDAC ameliorates various preclinical disease models that develop with age, including neurodegeneration, heart disorders, diabetes, and sarcopenia [31]. However, although vorinostat is the FDA-approved drug for patients with progressive, persistent, or recurrent cutaneous T-cell lymphoma (CTCL) following prior systemic therapies [32], it has not yet been studied for sarcopenia in humans. Based on the prior findings [31] and outcome of this study, we chose vorinostat for cellular experimental validation. C2C12 cells were treated with vorinostat (1 µM, 10 µM, 50 µM) for 24 h in a growth medium then transferred to a differentiation medium for 6 days. Our findings revealed that intervention with 1 µM of vorinostat increased the diameter of muscle fibers (Figure 8A,B,F). However, treatment with 10 µM and 50 µM of vorinostat significantly reduced cell proliferation, resulting in an insufficient local cell density for starting the differentiation of C2C12 cells (Figure S1A). Repeated WB experiments confirmed the drug intervention group (vorinostat: 1 µM) had a higher muscle fiber percentage (Figure 8C,D). The cell activity assays indicated vorinostat (1 µM, 2.5 µM, 5 µM, 10 µM) can inhibit the activity of C2C12 cells (Figure 8E). These results suggest that 1 µM of vorinostat may inhibit cell proliferation and promote differentiation of C2C12 cells while increasing the diameter of muscle fibers.

## 3. Discussion

Skeletal muscle is the largest organ of the human body and is primarily composed of muscle fibers that require a lot of energy to be contractile. The high energy demand of muscle requires a continual adenosine triphosphate (ATP) supply. Consequently, skeletal muscles are heavily dependent on mitochondria, the cellular powerhouses. However, mitochondrial function declines with age. Mitochondrial dysfunction in muscle would induce elevated apoptosis levels [38] and decreased regenerative capacity [39], which is a common pathological change in sarcopenic individuals [40]. Apart from changes in skeletal muscle function, age-related mitochondrial dysfunction can also lead to the death of motor neurons [41], causing impaired neuromuscular junctions and disabled muscle motor units, which could contribute to sarcopenia [42]. Our study revealed significant down-regulation of the “mitochondrial ATP synthesis coupled electron transport” and “NADH dehydrogenase complex assembly” pathways in individuals with sarcopenia, which may result in a decrease in ATP production, which is consistent with the previous discussion.

Recent research has indicated that disturbed mitochondrial function of muscle stem cells, instead of muscle fiber, can lead to sarcopenia [43]. The previous study [43] proposed that mitochondrial dysfunction of muscle fibers does not necessarily cause sarcopenia, as the resultant mitochondrial dysfunction can enhance the repair of damaged fibers, stimulating the differentiation of stem cells into muscle fibers. Conversely, mitochondrial dysfunction of muscle stem cells will impair the repair function of skeletal muscle which contributes to the development of sarcopenia. In this study, we found a significant decline in mitochondrial function among sarcopenia patients. All three drug repurposing approaches—which involved inverse of the expression of the differential genes, inverse of the expression of the hub genes, and the reverse enrichment of significant pathways—focused on drugs that could increase mitochondrial function. Moreover, the transcriptomic profiles treated with chemical compounds were derived from the SKB cell line (myoblasts), which is a muscle stem cell responsible for the growth, repair, and maintenance of skeletal muscle [44]. Taken together, these repurposing drugs that our integrated method recommends may be able to increase the mitochondrial function of SKB cells, thus effective in preventing and treating sarcopenia.

Vorinostat, a potent non-selective HDAC inhibitor, has earned approval from the US Food and Drug Administration (FDA) for treating cutaneous T-cell lymphoma. HDAC inhibitors play an important role in epigenetic control, and mounting evidence indicates that epigenetic modifications are closely associated with ageing [45,46]. Thus, histone deacetylase inhibitors have become promising medications to treat ageing-related conditions [31,47]. A previous study indicated vorinostat could enhance skeletal muscle mitochondrial function in vitro and in vivo [48]. Our study also indicated that 1 µM vorinostat could inhibit C2C12 cell proliferation and promote the formation of myotubes in vitro. However, 1 µM vorinostat does not significantly increase myotube formation (Figure S1B) when applied in a differentiation medium. The result was similar to a previous study [49]. This difference may result from hyperacetylation of MyoD, histones surrounding the MCK enhancer, and muscle-specific transcriptional activation [49]. Furthermore, vorinostat regulates cell cycle progression by inhibiting cell growth and promoting differentiation [50]. These results suggested that vorinostat may be a promising candidate to treat sarcopenia by promoting satellite and muscle stem cell differentiation.

The skeletal muscles are critical metabolic organs in the human body, playing a vital role in cytokines production and interactions with other organs [51]. Cytokines, adipokines, and myokines serve as critical mediators for such inter-organ crosstalk. As metabolic reprogramming mediators, myokines profoundly influence skeletal muscle performance and robustness [51]. Follistatin, a protein that binds with myostatin, exerts an inhibitory effect on myostatin and promotes muscle growth [52]. Studies carried out on both mice and humans have demonstrated the favorable influence of IL-6 in the regulation of muscle quality, insulin secretion, and lipid metabolism during physical exercise [53,54,55]. Both follistatin and IL-6 are myokines. Histone deacetylase (HDAC) is an important regulatory element involved in modulating skeletal muscle metabolism and enabling dynamic and adaptive response of muscles to various physiological and pathological circumstances [56]. The previous study has documented the interaction and mutual regulation between HDACs and myokines [51]. For example, HDAC4 regulates the secretion of myokines from skeletal muscle in response to injury, thereby modulating the myogenic potential and muscle regeneration of myogenic precursor cells (MPCs) [57]. Moreover, in malnourishment scenarios, pan-HDAC inhibitors (HDACi) have exhibited the efficiency of enhancing muscle regeneration by heightening the level of follistatin in muscle stem cells [58]. These findings suggest a close interaction between myokines and HDAC inhibitors. In this study, the potential of vorinostat in enhancing myotube formation highlights the need for a comprehensive understanding of its intricate regulatory relationship with myokines and skeletal muscle.

Our study may also have some limitations. First, our results are solely based on in vitro testing. We will further validate the therapeutic efficacy of vorinostat in aged mice, a common disease model for sarcopenia. Moreover, human-derived myoblasts serve as a superior experimental model for sarcopenia [59,60]; their use may yield a more accurate and reliable evidence base for the clinical translation of repurposed drugs. The etiology of sarcopenia is complex and not fully understood, with mitochondrial dysfunction, inflammation, satellite cell dysfunction, and oxidative stress, potentially contributing to sarcopenia risk [61]. In our analysis, probably due to the small sample size, sarcopenia-associated differential expression genes and hub genes were mainly enriched in mitochondrial-related pathways, thus the drugs repurposed from our recommendation algorithm may have a major effect on mitochondrial function. Further studies are required to determine whether repurposing drugs can be used against other underlying causes of sarcopenia. It is worth noting that vorinostat only enhances the formation of myotube in a growth medium instead of a differentiation medium. Analyzing the pharmacological effects of drugs at various cell growth stages will help us use them more accurately. Our future studies will also examine vorinostat’s effect on C2C12 at various developmental stages.

## 4. Methods

### 4.1. Data Collection

We conducted a transcriptomic analysis using two publicly available RNA-seq datasets in the Gene Expression Omnibus (GEO) database. The first dataset GSE111016 (bulk-RNA sequencing data) enrolled 40 male participants (20 healthy and 20 sarcopenic subjects), who were of Chinese descent and aged between 65 and 79 years. We utilized GSE111016 to compare the skeletal muscle transcriptome profiles of sarcopenic human subjects with those of healthy controls [15]. The second dataset GSE145480 (bulk-RNA sequencing data) contained mRNA profiles of gastrocnemius muscle from male C57BL/6JRj mice of different ages (8, 18, 22, 24, 26, and 28 months old), along with several functional indicators of skeletal muscle, such as grip strength, weight, and cross-sectional areas [22]. The GO_Biological_Process_2021 and KEGG_2019_Mouse were downloaded from Enrichr online database [62].

### 4.2. Gene Differential Analysis and Co-Expression Analysis

Differential gene expression was analyzed following the standard workflow of DESeq2 [63]. Raw counts of the GSE111016 and GSE145480 were used for differential analysis and significance was determined by FDR-adjusted *p*-values less than 0.05. WGCNA [64] was conducted on the two datasets to identify hub genes associated with sarcopenia. Raw read counts of all genes were transferred to TPM and the top 10,000 genes that ranked by median absolute deviation (MAD) were used for WGCNA. In the topological overlap measure (TOM) of gene modules, a threshold of 0.25 was used to merge similar gene modules with a minimum size of 30. The GSE111016 dataset was used to perform drug repurposing. The GSE145480 dataset (mouse) was used to demonstrate the repeatability of the result obtained from the differential analysis and WGCNA of the GSE111016 (human).

### 4.3. Drug Repurposing with Differential Analysis

The gene expression matrix of compounds was obtained from the Library of Integrated Network-based Cellular Signatures (LINCS) [65] database. LINCS [65] is a library that catalogs changes when different types of human cells are exposed to a variety of chemical compounds and genetic reagents (to overexpress or silence a specific gene). To identify drugs with potential therapeutic benefits for sarcopenia, we chose differential expression scores of SKB (myoblast) cell line as the characteristic signal of small molecules. Next, differential expression scores were arranged in descending order and transformed into a rank matrix (drug-gene matrix). The columns of the rank matrix represent small molecule compounds, the rows correspond to gene names, and the matrix contents indicate the rank value of the differential expression scores.

Drug repurposing was performed with the R package signatureSearch [25], which employed the differentially expressed genes from dataset GSE111016. The LINCS search algorithm, recommended in the signatureSearch [25], was used to filter drugs that might treat sarcopenia. The LINCS algorithm is one of the most efficient methods for drug repurposing [25]. It assigns weights to input genes according to differential expression scores of the drug-gene matrix for SKB cell line. A bi-directional weighted Kolmogorov-Smirnov enrichment statistic (ES) of input genes was used as a metric to rank repurposing drugs. Detailed documentation of the algorithm can be found in R package signatureSearch [25].

### 4.4. Drug Repurposing with Gene2drug

The intersection of hub genes within the WGCNA module and differential genes were designated as “significant genes”. The significant genes were used as input to perform drug repurposing using the gene2drug package [29]. Earlier, gene2drug was used to predict which genes could be up-regulated/down-regulated by drugs. In our study, we performed drug repurposing according to the number of “significant genes” that could be reverse regulated by the drug. If the drug can reverse-regulate more “significant genes”, the drug is more likely to be a potential treatment for sarcopenia.

### 4.5. Drug Repurposing with the Pathway Enrichment Analysis

Drug-gene enrichment analysis was performed using drug-induced gene expression profiles of the SKB cell line. For the enrichment analysis, we employed the gene set collections Gene Ontology (GO)-Biological Processes (BP) database as the gene set collection. The drug-induced gene expression profiles were transformed into a ranked pathway matrix [66]. To obtain the significant pathways associated with sarcopenia, we performed a ranked GSEA [20] based on the differential analysis of GSE111016. Subsequently, we computed the enrichment score for each drug to identify those that significantly up-regulate (or down-regulate) the significant pathways associated with sarcopenia. The top-ranked drugs were recommended based on the enrichment score.

### 4.6. Rank Aggregation

Marinka et al. [14] proposed the CRank algorithm utilized in our study. The CRank algorithm commences with the three ranked lists of drugs (Section 4.4, Section 4.5 and Section 4.6), accounts for uncertainty across ranked lists, and uses a two-stage iterative procedure to aggregate individual rankings. A detailed description of the algorithm can be found in the previous study [13].

### 4.7. Cell Culture and Treatments

To confirm the validity of the predictions, we performed mouse myoblast differentiation experiments. The Chinese Academy of Sciences provided the C2C12 mouse myoblasts. A Dulbecco’s Modified Eagles Medium (DMEM) containing 10% fetal bovine serum, 100 IU/mL penicillin, and 100 IU/mL streptomycin was used in the culture of C2C12 cells. Upon reaching 70–80% confluence, cells in T-25 flasks were regularly passaged. For drug intervention experiments, cells were seeded in six-well plates and grown for 24 h. Next, the culture medium was exchanged for a fresh growth medium (GM) containing the drug, and the cells were incubated for another 24 h. When the cells reached 90–100% confluence, the growth medium was switched into the differentiation medium, comprising DMEM supplemented with 2% horse serum, 100 IU/mL penicillin, and 100 IU/mL streptomycin. The differentiation process was continued for six days in DM to induce myoblast differentiation into myotubes, followed by harvesting for morphologic analysis and Western blotting. The culture medium was changed at least once every two days. In this study, we tested the effects of vorinostat at 1 µM, 10 µM, and 50 µM concentrations on C2C12.

### 4.8. Measurement of Cell Diameter

To assess whether candidate drugs influence C2C12 differentiation, the diameter of single myotubes were measured. The differentiated myotubes were photographed with an inverted microscope. Each well of the six-well plate was divided into five areas and randomly photographed myotubes in each quadrant area under 20× magnification. The myotubular map diameters were processed using ImageJ software. The middle part of the myotubes with a more uniform diameter size was selected for myotube size measurement. Five images were taken for each well, and finally 15 images for each of the drug intervention or control groups were included in the cell diameter analysis.

### 4.9. Western Blotting (WB)

To ascertain changes in myotube quantity or size following pharmacological intervention, we performed western blotting using the following procedures. Myotubes in a six-well plate were washed thrice in pre-cooled PBS solution before lysed with 100 μL of the radioimmunoprecipitation assay (RIPA) buffer (NCM Biotech, Soochow, China) and ultra-sonication. Cell debris was removed by centrifugation at 12,000 rpm for 30 min. Subsequently, protein samples were mixed with 5X SDS-PAGE Sample Loading Buffer, boiled for 20 min, then centrifuged at a rate of 12,000 rpm for 15 min. SDS-PAGE was performed using 4–12% FuturePAGE precast protein gels (ACE Biotechnology, Nanjing, China) at 160 v for 40 min, then protein samples were transferred to PVDF (Immobilon™-P) membranes at 400 mA for 40 min. The membrane was blocked with NcmBlot blocking buffer (NCM Biotech, Soochow, China) for 10 min at 37 °C. Thereafter, we incubated the membranes with primary antibodies (β-actin (1:10,000; cat. no AF7018), Myh2 (1:10,000; cat. no. ab124937)) at 4 °C overnight then used anti-rabbit IgG as the secondary antibodies. All antibodies were diluted in NCM Universal Antibody Diluent (NCM Biotech, Soochow, China). Secondary antibodies were incubated with membranes at 37 °C for 60 min. Tween 20 (PBST) was used to wash the membranes. Chemiluminescent Imaging was visualized using QuickChemi5200 (Monad, Wuhan, China).

## Figures and Tables

**Table 1:** Five drugs/small molecules may have a therapeutic effect on sarcopenia.

Drug	Target	CRank	D1	D2	D3	Reference Article
Danazol	AR; CCL2; CYP2C8; ESR1; GNRHR; GNRHR2; PGR; PLG; PROS1; SERPINA6; SERPINC1; SERPING1; SHBG; TNF	5	67	1	6	Danazol increases lean tissue mass [33]
estradiol-benzoate	ESR1	6	18	23	41	Estrogen recovers exercise endurance in female mice [34]
SB-431542	ACVR1B; ACVR1C; TGFBR1	8	2	11	109	SB-431542 could increase Human pluripotent stem cells myotube fusion [35]
diclofenac	AKR1C3; ALOX5; ASIC1; ASIC3; CYP2C8; CYP2C9; KCNQ2; KCNQ3; LTF; PLA2G2A; PPARG; SCN4A; TF; TNF; ZADH2	11	61	60	30	The utilization of NSAIDs revealed a decreased susceptibility to sarcopenia in users as compared to non-users. (OR 0.26, 95% CI: 0.08–0.81) [36]
budesonide	BGLAP; CCL11; CCL5; CSF2; CYP3A5; CYP3A7; ICAM1; IL4; IL5; IL8; NR3C1	16	27	53	102	Budesonide promotes the terminal differentiation of satellite cells [37]

Note: D1 represents the rank of drug repurposing with the differential analysis; D2 represents the rank of drug repurposing with the gene2drug algorithm; D3 represents the rank of drug repurposing with pathway enrichment analysis.

## Data Availability

The data presented in this study are openly available in GEO (Gene Expression Omnibus) at [10.1038/s42003-021-01723-z] and [10.1038/s41467-019-13694-1]. The reference number included [GSE145480] and [GSE111016]. Data is contained within the article and Supplementary Materials.

## Supplementary Materials

The following supporting information can be downloaded at: https://www.mdpi.com/article/10.3390/ph16040607/s1, Figure S1: (A) 10 µM and 50 µM of vorinostat significantly reduced cell proliferation; (B) 1 µM of vorinostat does not increase the diameter of muscle fibers in the differentiation medium; Table S1: 222 differentially expressed genes of GSE111016; Table S2: Green module gene; Table S3: The result of drug repurposing with LINCS algorithm; Table S4: Sarcopenia-associated genes were obtained from the Open Targets and the DisGeNET database; Table S5: The result of drug repurposing with gene2drug algorithm; Table S6: The result of drug repurposing with pathway2drug algorithm; Table S7: The result of the Crank algorithm.
